# Multiple liver masses in a patient with breast cancer, metastasis or not? An unexpected diagnosis: hepatic fascioliasis. A case report and overview of the literature from Turkey

**DOI:** 10.1259/bjrcr.20160038

**Published:** 2016-05-08

**Authors:** Çağri Damar, Arif Emre Emek, Hüseyin Uçar, Harun Erdal, Işik Conkbayir, Çiğdem Öztunali

**Affiliations:** ^1^Department of Radiology, Gazi University, Ankara, Turkey; ^2^Department of Radiolology, Civril Government Hospital, Denizli, Turkey; ^3^Department of Radiology, Afyon Kocatepe University, Afyon, Turkey; ^4^Department of Gastroenterology, Gazi University, Ankara, Turkey; ^5^Department of Radiology, Diskapi Yildirim Beyazit Education And Research Hospital, Ankara, Turkey

## Abstract

A patient who underwent mastectomy of the left breast owing to breast cancer was referred to our department for abdominal ultrasonography during her routine check-up. Radiological examinations demonstrated multiple masses that tended to form clusters in the liver parenchyma, and the lesions were initially thought to represent metastases from the breast cancer. Multisite biopsies and serological tests confirmed the diagnosis of *Fasciola hepatica* infestation. To our knowledge, this is a unique case report of a patient with a known malignant neoplasm. We also present an overview of the literature about human fascioliasis in Turkey.

## Summary

Owing to their long historical background, environmental and climatic features, biodiversity and proximity of the people to animals some zoonotic diseases are seen more frequently in the countries of the Mediterranean and the Middle East.^1^ *Fasciola hepatica* is a foodborne trematode worm (fluke) that mainly affects the liver of its final host. Its human infestation can be seen in Anatolia (the western peninsula of Asia that forms the mainland of Turkey). As its radiological findings may mimic metastases, it is important to consider *F. hepatica* in the differential diagnosis while evaluating imaging findings of patients with previously known malignancy. We herein present the case of a 38-year-old female patient with a history of breast cancer who had presented with multiple liver lesions that were finally proven to be consistent with fascioliasis.

## Case Report

A 38-year-old female who had undergone right mastectomy for breast carcinoma was referred to our department for evaluation of the multiple liver masses detected on routine follow-up examinations.

**Figure 1.**
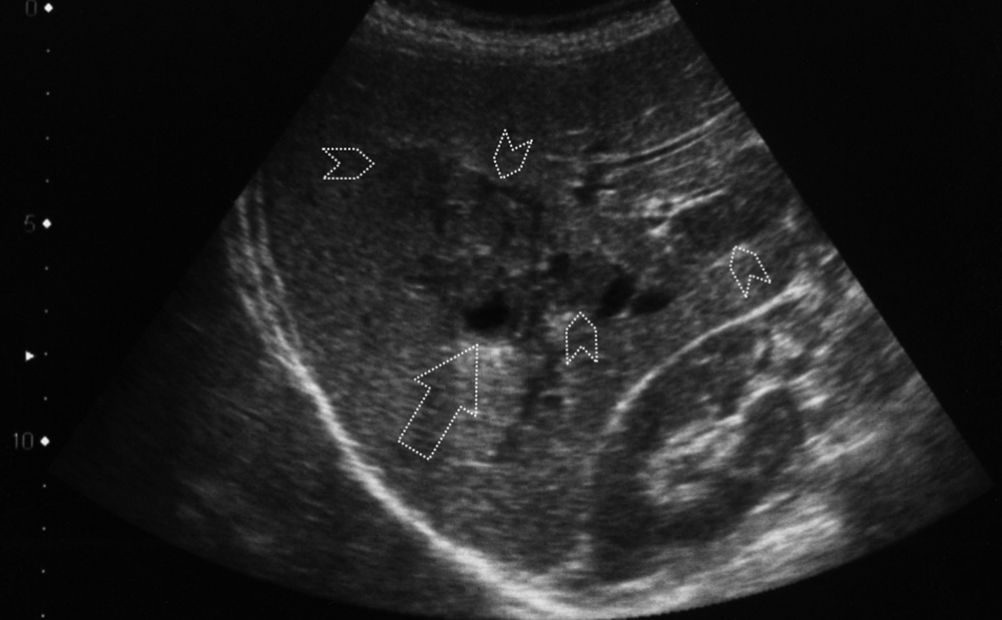
Ultrasound image of the liver shows multiple, ill-defined hypoechoic confluent nodular masses (chevron arrows). Note the central necrosis area in one of the larger-sized lesions (large arrow).

**Figure 2.**
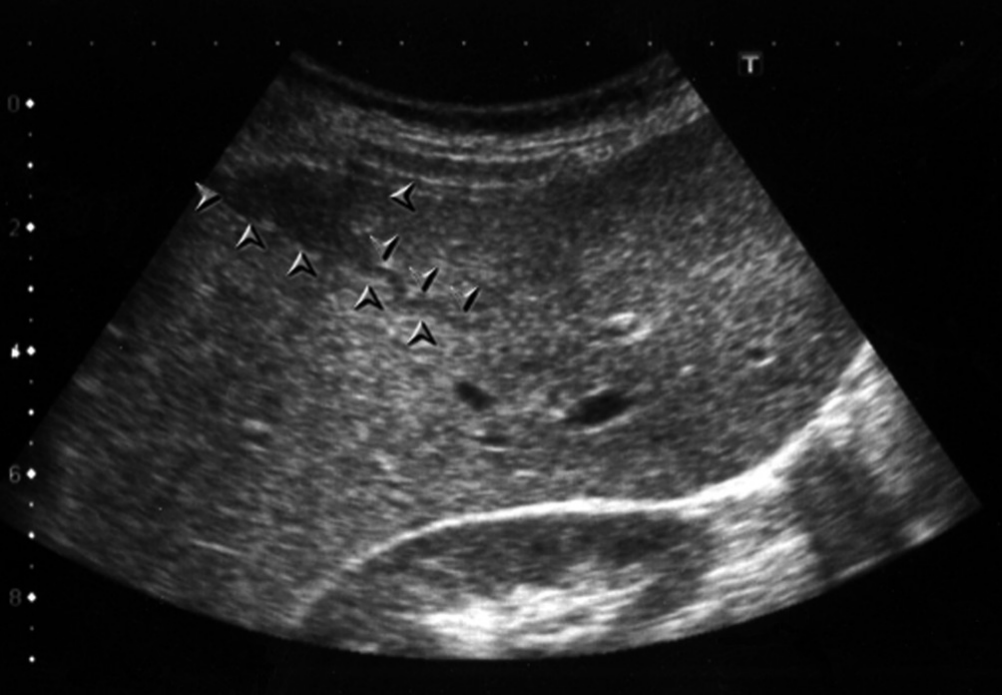
Ultrasound image of the liver reveals poorly defined hypoechoic subcapsular area and a tortuous tract, extending from the capsule to the deeper parenchyma (arrowheads).

Sonographic examination of the patient revealed hepatosplenomegaly and multiple heterogeneous hypoechoic masses of varying sizes that were located centrally in the right lobe of the liver and tended to form clusters. Some of the larger lesions had central necrosis and cavitation areas (Figure 1). We observed hypoechoic curvilinear tracts extending from the liver capsule to the parenchyma, which did not demonstrate abnormal vascularity on colour Doppler ultrasound examination (Figure 2). The gallbladder had normal wall thickness and endoluminal echogenicity.

**Figure 3.**
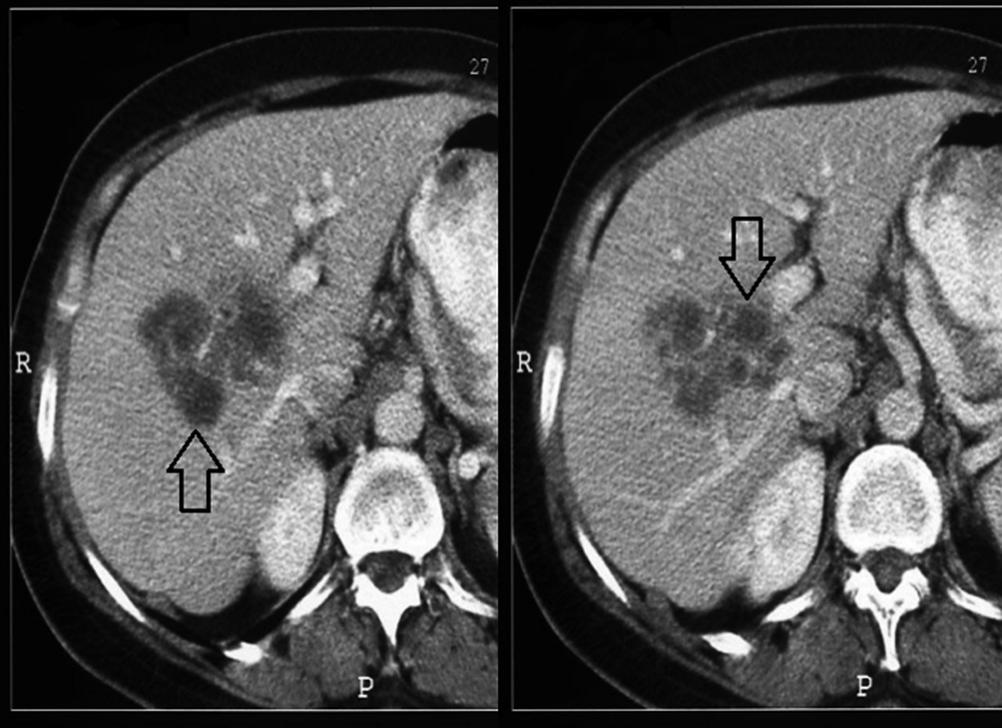
Contrast-enhanced CT images in the portal phase show multiple small, clustered hypodense nodular masses with hazy irregular margins in the right lobe of the liver parenchyma (arrows).

**Figure 4.**
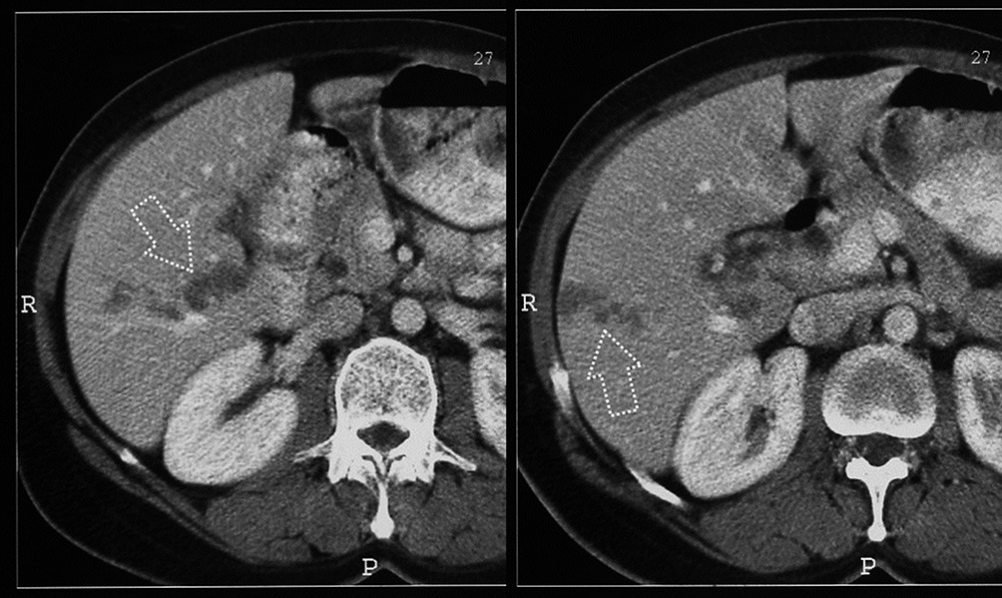
Contrast-enhanced CT images in the portal phase demonstrate two hypodense curvilinear tracts, extending from the liver capsule to the parenchyma, in the hepatic segments V and VI (arrows).

Contrast-enhanced CT obtained in the portal venous phase revealed multiple, clustered, hypodense nodular liver masses with irregular margins (Figure 3). In addition, the presence of at least three hypodense curvilinear tracts, extending from the liver capsule to the parenchyma, were confirmed (Figure 4). The lesions did not demonstrate any contrast enhancement. There were a number of enlarged lymph nodes in the porta hepatis.

**Figure 5.**
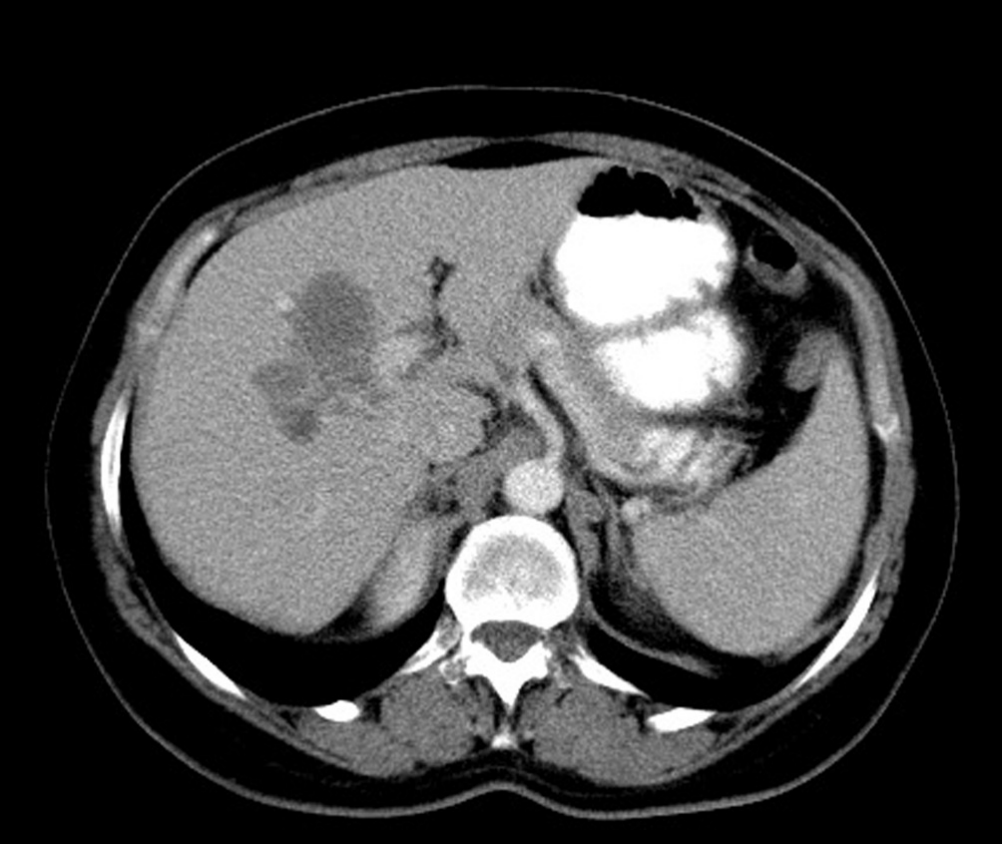
Post-treatment contrast-enhanced CT image in the portal phase 2 years later shows that the liver lesions had slightly regressed in size.

Ultrasound-guided fine needle aspirations of the selected lesions were performed. Pathological examination of the specimens showed no evidence of malignancy but eosinophil-rich inflammatory necrotic tissue was reported. Blood tests also revealed elevated levels of eosinophils and serological tests confirmed the presence of *F. hepatica* infestation. The patient was discharged after a course of triclabendazole treatment. A follow-up CT scan, obtained 2 years after her first admission, showed slight regression in the size of all the liver lesions (Figure 5 and Supplementary Video A and there were no new lesions.

## Discussion

Fascioliasis is included in the group of food (or snail)-borne trematodiases and is caused by two species of parasitic flatworms: *F. hepatica* and *Fasciola gigantica.*^2^

### Epidemiology

Although the natural final hosts of this parasite are sheep, goats, cattles and other herbivores, *F. hepatica* has been reported in humans with increasing frequency. Despite the belief that it has a European origin, *F.hepatica* has shown a great capacity to spread and can be seen in all the continents except Antarctica. According to the recent estimates, several million people are infected in more than 70 countries worldwide, with millions of others being at risk. The parasite can easily adapt and colonize in rural areas and wetlands, where there are plenty of freshwater *Lymnaea* snails (the intermediate hosts) and herbivorous mammals (the definitive hosts). However, human fascioliasis (HF) has a patchy geographical distribution and there are some endemic regions such as the Caribbean, northern Africa, western Europe and the Caspia. Certainly, the lifestyle and dietary habits of the people, social and economic factors, hygiene and sanitation conditions, and travel or immigration, all add to the frequency of the disease. If it is detected in some livestock animals, human cases may also exist.^2–6^

### Lifecycle of parasites

The eggs of the parasite are excreted with the final host's faeces. These eggs are immersed in freshwater, and the swimming, ciliated miracidia forms are released. Miracidiae use *Lymnaea* snails as their first host; once they metamorphose into motile, tailed cercaria forms, they leave the snails. Cercariae use the aquatic plants (watercress, watermint, lettuce, spinach orother salad vegetables) as another group of intermediate hosts; the cercarial larvae encyst and develop into the metacercarial stage. The main transmission of *F. hepatica* occurs by ingesting water or raw plants contaminated by the metacercaria forms of the parasite. The metacercariae change form during duodenal interference; they penetrate the intestinal wall and migrate through the peritoneal cavity to reach the Glisson’s capsule. After piercing the capsule of the liver, the parasites migrate through the liver parenchyma and reach the biliary system, where they grow into adults and release new eggs. The eggs reach the intestines *via* bile and are evacuated in the faeces, thus completing the transmission cycle of the parasites. The average size of a mature fluke is 20–40 mm in length and 8–13 mm in width.^6–8^

### Human infestation, clinical features, diagnosis and treatment

Human infestation by this trematode has two characteristic phases: an acute *hepatic (parenchymal) phase* and a chronic *biliary (ductal) phase*.^6,7^

The first phase is the hepatic phase. In this phase, the parasites pierce the Glisson’s capsule and migrate through the liver parenchyma towards the bile ducts in a random manner. This stage lasts for 1–3 months after metacercariae infection. The clinical features of this stage include anorexia, fatigue, nausea, vomiting, right upper quadrant pain, pruritus, fever, weight loss, respiratory symptoms, hepatomegaly, jaundice and urticaria. Laboratory findings such as elevated liver enzymes, increased erythrocyte sedimentation rate, hypergammaglobulinaemia and marked eosinophilia can be encountered in this stage as well. Mild hepatitis, severe hepatic subcapsular haemorrhage or liver necrosis may also be seen rarely in this hepatic phase.^6,7^

The second stage may be asymptomatic for a long time or may be characterized by intermittent right upper quadrant pain. As a result of chronic inflammation of the bile ducts, ductal wall thickening, common bile duct obstruction, bile duct stones or gallstones, cholestasis, cholangitis, cholecystitis or pancreatitis may occur.^6,7,9^ Adult flukes can live for years and the chronic phase may persist in untreated patients.^8,10^ *F. hepatica* can rarely settle in unusual organs and lead to ectopic disease.^8,11–13^

Suggested diagnostic criteria for fascioliasis in endemic regions, reported by the WHO in 2009, are:^14^

- history of eating raw aquatic plants
- clinical symptoms such as abdominal pain in the epigastric or right-upper quadrant region, lasting at least a week
- eosinophilia
- positive ultrasound or CT scan findings
- positive for the presence of *F. hepatica* eggs (detected by Kato–Katz thick smear or sedimentation technique)
- positive serologic tests (from the serum, stool or urine specimens).

For the treatment of the cases diagnosed with the above-mentioned criteria, the recommended drug triclabendazole (10 mg kg^−1^ of body weight) is given in a single administration. If necessary, a double dose (20 mg kg^−1^ of body weight) can be administered for the management of individual cases.^14^

### Radiological findings

When the flukes follow the migration path from the liver capsule to the bile ducts, they ingest hepatocytes. Along this migration path, multiple small necrotic cavities and microabscesses arise. These abscess cavities are not large, as seen in the cases of other suppurative processes. On CT imaging, migration paths that extend from the Glisson's capsule to deep parenchymal areas (tunnel-like tracts) can be seen. Also, serpentine, clustered, small necrotic cavities can be detected. These two findings, which were also observed in our case, are defined as the “tunnels and caves sign” in the literature^10^ and can help in separating other malignant lesions from these parasitic lesions. In addition, the peripheral halo sign on B-mode ultrasound examination and internal vascularity on colour Doppler mode sonography or contrast-enhanced CT imaging, which are usually seen in cases of metastatic nodules, were not present in our case.^15^

**Figure 6.**
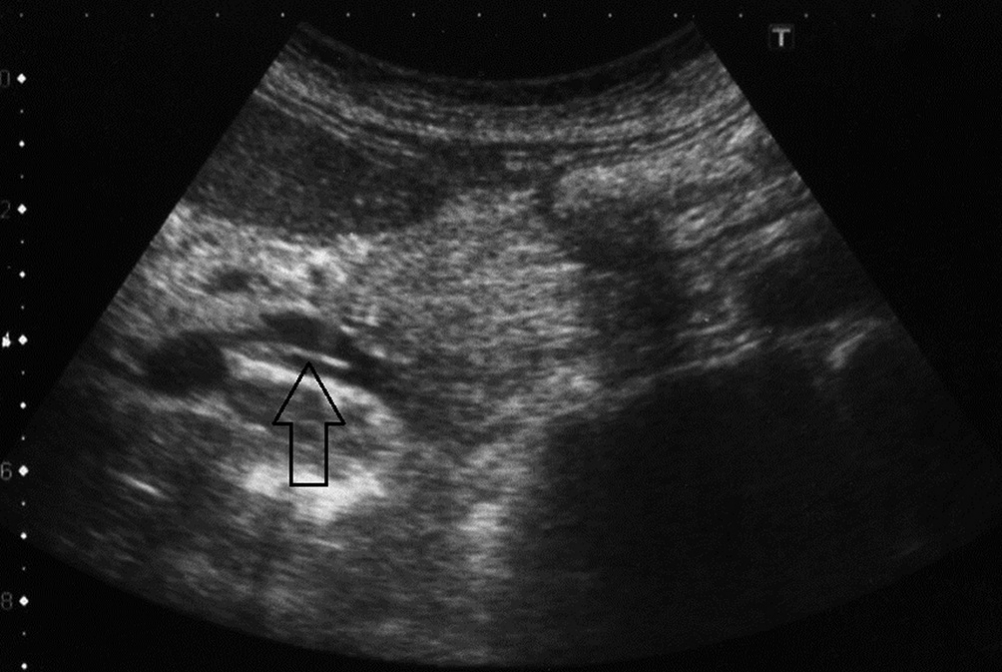
Ultrasound image reveals a thin, linear echogenicity, which is fixed at one end to the posterior wall of the common bile duct.

With the use of ultrasound imaging, adult worms can be visualized in the gallbladder and/or in the extrahepatic bile ducts. Ultrasound examination may demonstrate single or multiple, elongated filamentous structures or moving echogenic flukes.^10^ Adult forms of parasites attach to the inner wall of the bile duct through their ventral suckers.^9,10^  When we retrospectively re-examined our patient, we detected a thin, linear echogenicity 1.5 cm long, one end of which was fixed to the posterior wall of the common bile duct (Figure 6). Although this appearance was also consistent with the previously described sonographic appearances of the adult parasites in the literature,^8,16^ we did not perform any further investigations because the patient had already been diagnosed with the infection and there were no signs of cholestasis that would have required further endoscopic intervention. There are also some reports that stress the importance of endoscopic retrograde cholangiopancreatography (ERCP) in the diagnosis and treatment of the chronic phase of the disease. In cases of cholestasis, adult parasites can be seen in the biliary tract and ERCP enables the removal of the parasites.^17^ In ERCP or MR cholangiography, the flukes are seen as filling defects in the bile ducts.^8,17,18^

Periportal lymphadenopathies may be accompanied by other radiological findings, as in our case.^15^

Other cases of hepatobiliary fascioliasis mimicking cholangiocellular or hepatocelluler carcinoma or Oddi sphincter malignancy have been reported. Extrahepatic fascioliasis mimicking colon, ovarian, peritoneal carcinoma, or liver metastases of unknown origin have also been reported in the literature.^19–27^ However, only one case of hepatic fascioliasis has been reported in a patient with breast carcinoma by Koc et al^28^ in 2009. The patient was asymptomatic and only choledochal dilatation was found incidentally during abdominal ultrasound examination; without any other suspicious imaging findings of metastasis. To our knowledge, except for our report, there is no other report of HF mimicking liver metastases in a patient being followed up for breast carcinoma.

#### HF in Turkey

HF is a re-emerging disease and common in some provinces of Turkey^29^ (Table 1). There are many published reports of human FH infections coming from these regions (Table 2), and also there are some case reports related to European travellers.^30–33^* F. gigantica* does not have the ability to spread like *F. hepatica*, but can be seen infrequently in Turkey.^4^ There are only two case reports of biliary *F. gigantica*, which is distinguished from *F. hepatica* with its morphological features.^34,35^

The disease continues to attract a large number of physicians who are studying in different departments in Turkey.

**Table 1.** Publications reporting the prevalence of human fascioliasis (HF) in some regions of Turkey

Author (year)	City/ Region	Number of selected individuals	Number of detected HF	Prevalence	
				Serological tests (%)	Stool tests (%)
Yilmaz et al^36^ (1997)	Van/Eastern Anatolia	3.534^*a*^ (14 years and above)	1		0.028
Yilmaz et al^37^ (1998)	Ercis (Van)/Eastern Anatolia	206*^a^* (students, 7–15 years age group)	5		2.43
Yilmaz et al^38^ (1999)	Ercis (Van)/Eastern Anatolia	293*^a^*	2		0.68
Yilmaz et al^39^ (2004)	Ercis (Van)/Eastern Anatolia	500	9		1.8
Yilmaz et al^40^ (2007)	Ercis (Van)/Eastern Anatolia	867*^a^*	1		0,1
Tas Cengiz et al^41^ (2009)	Van/Eastern Anatolia	2.975*^a^*	1		0.03
Tas Cengiz et al^42^ (2015)	Van/Eastern Anatolia	1.600	89	5.6	1,8
Tas Cengiz et al^43^ (2015)	Van/Eastern Anatolia	5.985*^a^*	8		0.1
Sener et al^44^ (1998)	Ankara/Central Anatolia	122.400*^a^*	1		0.0009
Kaplan et al^45^ (2002)	Elazıg/Eastern Anatolia	540	15	2.78	
Seker et. al^46^ (2005)	Bagtepe (Adana)/Mediterranean	291	30	10.3	
Turhan et al^47^ (2006)	Antalya (10 suburbs)/Mediterranean	597	18	3	
Demirci et al^48^ (2003)	Isparta/Mediterranean	756, with eosinophilia	46	6.1	
		320, others	3	0.9	
Kaya et al^49^ (2006)	Isparta/Mediterranean	415	10	2.4	
	A. Gokdere (Isparta)/Mediterranean	171	16	9.3	
Ozturhan et al^50^ (2009)	Mersin/Mediterranean	155, with a family history of HF	3	1.93	
		729, others	4	0.55	
Sahin et al ^51^ (2008)	Karpuzeskisi (Kayseri)/Central Anatolia	374	13	3.48	
Koksal et al^52^ (2010)	Istanbul/Marmara	27.664*^a^*	1		0.003
Zeren et al^53^ (2013)	Cukurova (Adana)/ Mediterranean	94, with blood samples obtained during forensic autopsies	13	13.8	
Tas et al^54^ (2014)	Bolu/Black Sea	2.595*^a^*	1		0.039
Karaman et al^55^ (2014)	Ordu/Black Sea	7.194*^a^*	17	0.23	

*^a^*The study includes not only HF but also other parasitoses.

**Table 2.** The other publications on human fascioliasis from Turkey, including at least five or more cases

Author (year)	Hospital/department of the corresponding author	City/region	Number of cases	Date range
Kabaalioglu et al^15^ (2007)	Akdeniz UMH/radiology	Antalya/Mediterranean	87^*a*^	1995–2006
Saba et al^56^ (2004)	Akdeniz UMH and other centers/infectious siseases	Antalya/Mediterranean	53	1998– 2003
Cevikol et al^57^ (2003)	Akdeniz UMH/radiology	Antalya/Mediterranean	43	1995– 2001
Cubuk et al^58^ (2001)	Akdeniz UMH/radiology	Antalya/Mediterranean	52	1995– 2000
Sakru et al^59^ (2004)	Trakya UMH and other centers/ microbiology	İzmir, Antalya/Aegean, Mediterranean	37*^b^*	?–2004
Taylan Ozkan et al^60^ (2005)	Ege UMH and other centers/parasitology	İzmir, Antalya/Aegean, Mediterranean	14	?–2005
Sezgi et al^61^ (2013)	Dicle UMH/pulmonary disease	Diyarbakir/South-Eastern Anatolia	56	2010–2011
Teke et al^62^ (2014)	Dicle UMH/radiology	Diyarbakir/South-Eastern Anatolia	45	2011–2013
Basarili et al^63^ (2011)	Dicle UMH/biochemistry	Diyarbakir/South-Eastern Anatolia	45	2010–2011
Kaya et al^64^ (2013)	Dicle UMH/gastroenterology	Diyarbakir/South-Eastern Anatolia	42*^c^*	2010–2012
Ulger et al^65^ (2014)	Dicle UMH/general surgery	Diyarbakir/South- Eastern Anatolia	39	2005–2013
Demirkaya et al^66^ (2014)	Dicle UMH/microbiology	Diyarbakir/South-Eastern Anatolia	13	2011–2012
Demirci et al^67^ (2009)	Suleyman Demirel UMH/microbiology	Isparta/Mediterranean	50*^d^*	?–2009
Yesildag et al^16^ (2009)	Suleyman Demirel UMH/radiology	Isparta/Mediterranean	27	2001–2006
Avcu et al^18^ (2009)	Van Yuzuncu Yil UMH/radiology	Van/Eastern Anatolia	24	2008
Karahocagil et al^68^ (2011)	Van Yuzuncu Yil UMH/infectious disease	Van/Eastern Anatolia	24	2008
Aksoy et al^69^ (2006)	Hacettepe UMH/internal medicine	Ankara/Central Anatolia	10	1998–2005
Karadag-Oncel et al^70^ (2012)	Hacettepe UMH/pediatric infectious disease	Ankara/Central Anatolia	5	2005–2011
Kayabalı et al^71^ (1992)	Ankara UMH/general surgery	Ankara/Central Anatolia	7	?–1992
Tezer et al^72^ (2013)	Gazi UMH and other centers/pediatric infectious disease	Ankara/Central Anatolia	6	2008–2012
Tetik et al^73^ (1995)	Ankara Diskapi ERH/general surgery	Ankara/Central Anatolia	5	?–1995
Sezgin et al^74^ (2004)	Mersin UMH and other centers/gastroenterology	Mersin/Mediterranean	9	1996–2002
Parsak et al^75^ (2006)	Cukurova UMH/general surgery	Adana/Mediterranean	10	2000–2006
Koc et al^28^ (2009)	Baskent UMH/radiology	Adana/Mediterranean	5	2003–2007
Gulsen et al^76^ (2006)	Gaziantep UMH/internal medicine	Gaziantep/South-Eastern Anatolia	5	2000–2003

ERH, Education and Research Hospital; UMH, University Medical Hospital.

*^a^*The largest case series described in one of the five publications (1999–2013) of the author.

*^b^*The largest case series described in one of the two publications (2004, 2011) of the author.

*^c^*The largest case series described in one of the three publications (2011–13) of the author.

^*d*^The largest case series described in one of the seven publications (2002–09) of the author.

Note: The published case series from the same centres may overlap.

## Conclusions

Hepatobiliary fascioliasis should be kept in mind in the differential diagnosis of multiple hepatic masses when suggestive clinical and laboratory findings are present, especially when the patients come from the endemic regions. Knowledge of the radiological imaging characteristics of HF can aid in the diagnosis, and imaging is also useful in the evaluation of the treatment response.

## Learning Points

1. *F. hepatica* infestation must be considered in case of patients with a history of eating raw aquatic plants (watercress, etc.) in endemic regions who present with right upper quadrant pain, eosinophilia and multiple hepatic lesions.
2. Hepatobiliary fascioliasis can mimic primary or secondary hepatic malignancies.
3. “Tunnels and caves sign” is a finding of HF in radiological examinations.

## Consent

Written informed consent was obtained from the patient for publication of this case report, including accompanying images.
